# Supplementary material for: Endogenous BAX and BAK form mosaic rings of variable size and composition on apoptotic mitochondria
Source: Cell Death Differ. 2024 Mar 19;31(4):469–78. doi: 10.1038/s41418-024-01273-x (PMC11043412; doi:10.1038/s41418-024-01273-x)
Supplement: Supplementary file 1 — Supplemental Material [file 41418_2024_1273_MOESM1_ESM.pdf]

Supplementary Information

Endogenous BAX and BAK form mosaic rings of variable size and composition on apoptotic mitochondria

Sarah V. Schweighofer et al.

FigS1

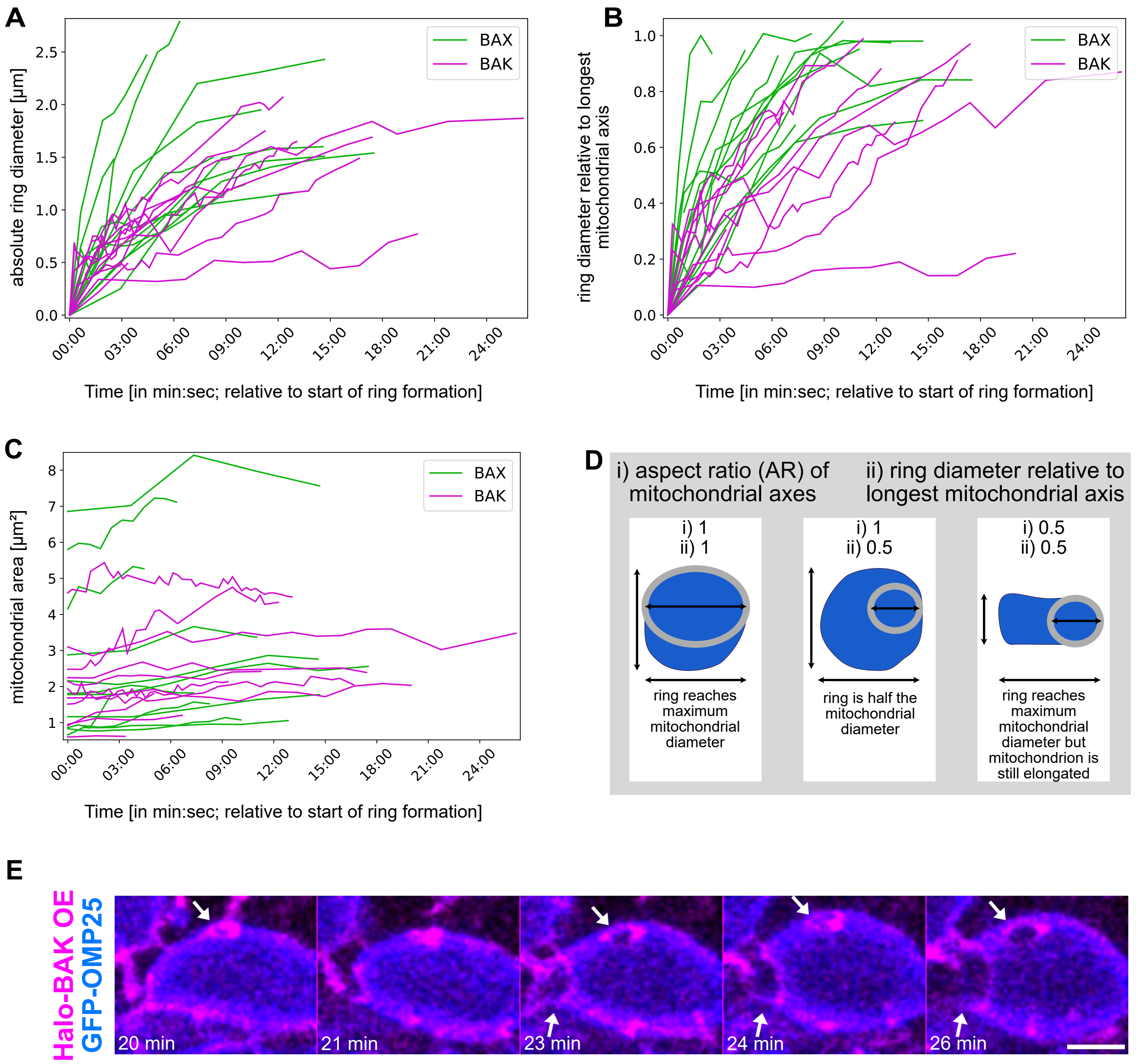
A
absolute ring diameter [µm]
Time [in min:sec; relative to start of ring formation]
BAX
BAK
B
ring diameter relative to longest mitochondrial axis
Time [in min:sec; relative to start of ring formation]
BAX
BAK
C
mitochondrial area [µm²]
Time [in min:sec; relative to start of ring formation]
BAX
BAK
D
i) aspect ratio (AR) of mitochondrial axes
ii) ring diameter relative to longest mitochondrial axis
i) 1
ii) 1
ring reaches maximum mitochondrial diameter
i) 1
ii) 0.5
ring is half the mitochondrial diameter
i) 0.5
ii) 0.5
ring reaches maximum mitochondrial diameter but mitochondrion is still elongated
E
Halo-BAK OE
GFP-OMP25
20 min
21 min
23 min
24 min
26 min

FigS1. Differences in BAX and BAK pore formation.

(A) Absolute ring diameter of BAX (green) or BAK (magenta) rings plotted over time.

(B) Relative ring diameter (to longest mitochondrial axis) of BAX (green) or BAK (magenta) rings plotted over time.

(C) Mitochondrial area of the mitochondrial fragments harboring BAX (green) or BAK (magenta) rings plotted over time.

(A-C) Timepoint 00:00 is relative and individually corresponds to the frame in which the ring formation of each analyzed ring started.

(D) Schematic illustration of AR (i) and relative ring diameter (ii).

(E) Selected frames of a live-cell STED movie from a U-2 OS WT cell undergoing apoptosis. The cells stably overexpressed GFP-OMP25 (blue, confocal imaging mode) and were transiently transfected with Halo-BAK (labeled with Atto590, magenta). Cells were imaged in the presence of 20 µM QVD-OPh. Two BAK rings form on one mitochondrial fragment (arrows). Timepoints correspond to the frames of the movie.

Data are quantified from 23 individual rings in 3 biological replicates of 2 independent experiments per condition.

Scale bar: 1 µm (E).

FigS2

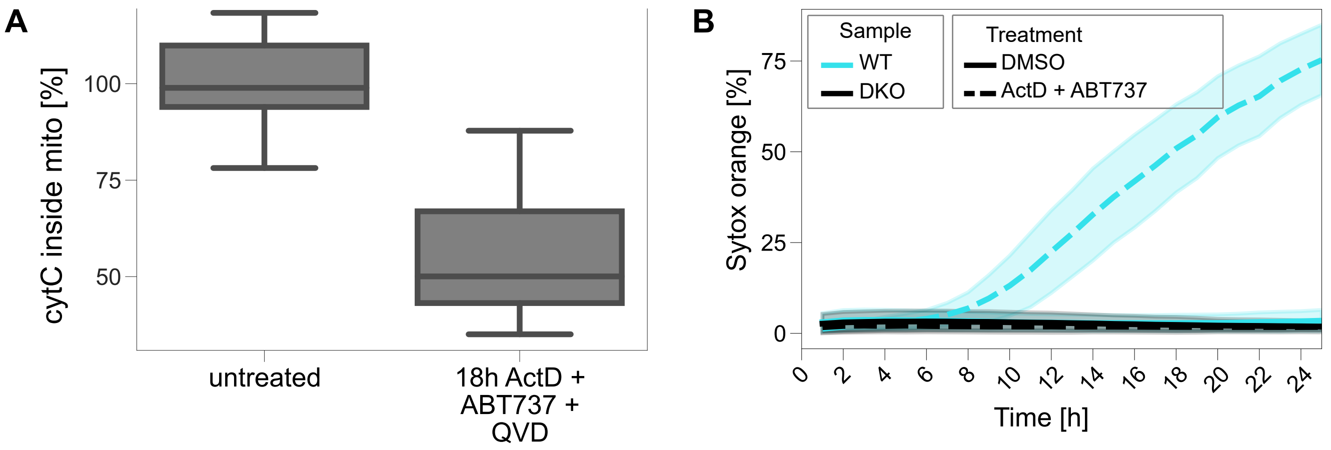
A
cytC inside mito [%]
100
75
50
untreated
18h ActD + ABT737 + QVD
B
Sample
WT
DKO
Treatment
DMSO
ActD + ABT737
Sytox orange [%]
75
50
25
0
0
2
4
6
8
10
12
14
16
18
20
22
24
Time [h]

FigS2. Cell death assessment.

(A) Quantification of the fluorescence intensity of cytochrome *c* inside the mitochondria (mito) of U-2 OS WT cells. Untreated cells or cells treated with 10 µM ABT-737 and 10 µM Actinomycin D as well as 20 µM QVD-OPh for 18 h were fixed and immunolabeled for TOM20 and cytochrome *c*.

(B) Quantification of percentage of cells with permeable plasma membrane (Sytox orange uptake) in U-2 OS WT cells compared to BAX-BAK-double-KO cells ("DKO") under treatment or under DMSO (control). Cells were imaged live over 24 h in the presence of Hoechst and Sytox Orange.

FigS3

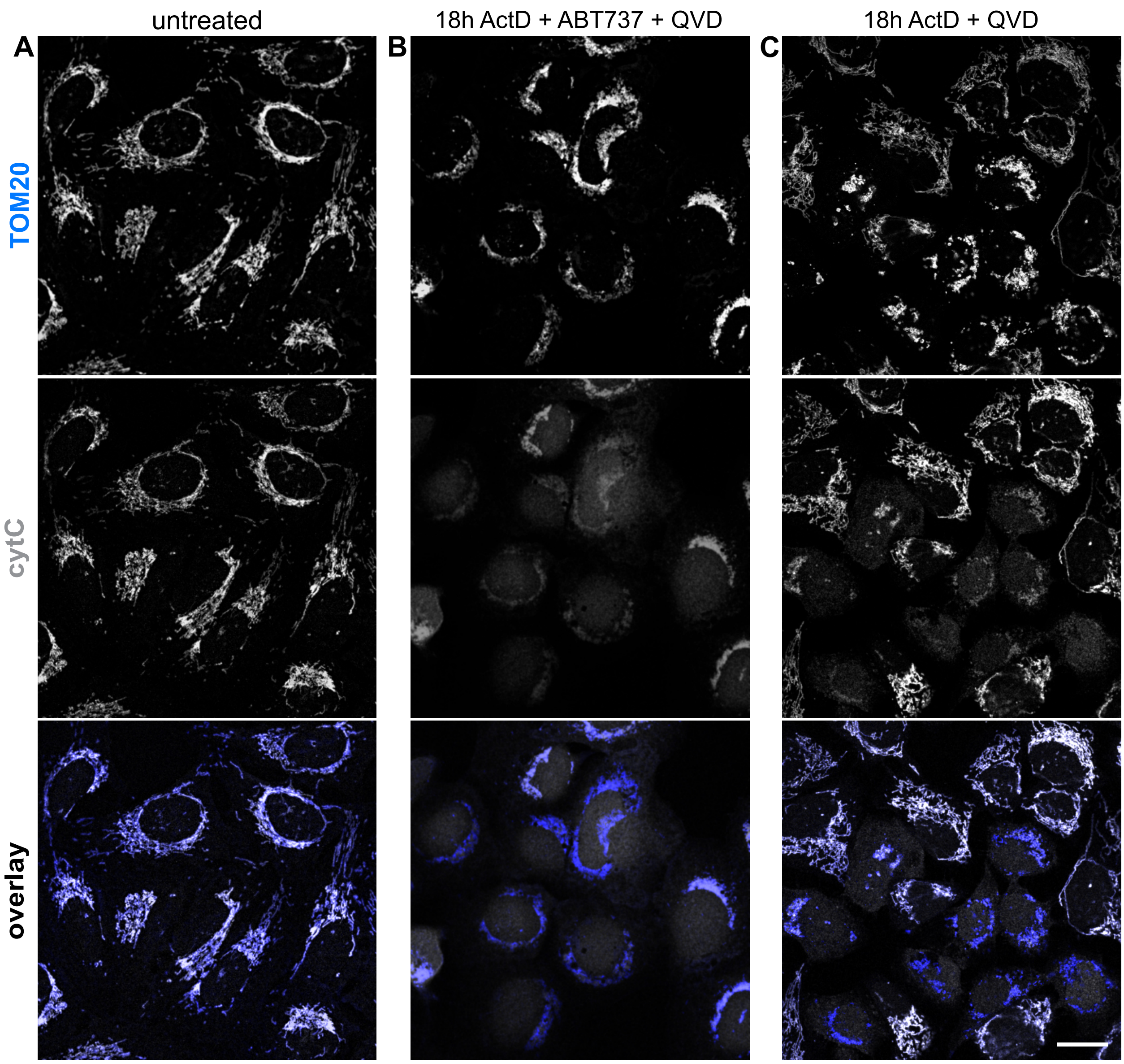
untreated
18h ActD + ABT737 + QVD
18h ActD + QVD
A
B
C
TOM20
cytC
overlay

FigS3. Cytochrome *c* release under treatment.

Confocal images of fixed U-2 OS cells, immunolabeled for TOM20 (blue) and cytochrome *c* ("cytC", gray). Cells were **(A)** untreated, **(B)** treated for 18h with 10 µM ABT-737, 10 µM Actinomycin D and 20 µM QVD-OPh or **(C)** treated for 18h with 10 µM Actinomycin D and 20 µM QVD-OPh, without the addition of ABT-737.

Scale bar: 20 µm

FigS4

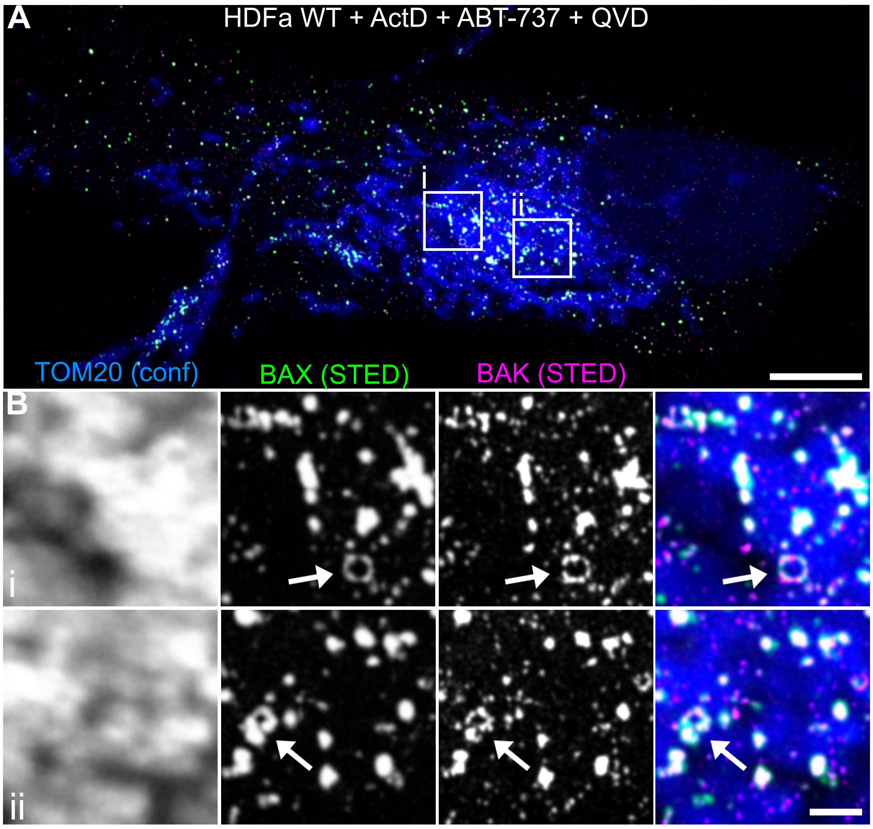
A
HDFa WT + ActD + ABT-737 + QVD
i
ii
TOM20 (conf)
BAX (STED)
BAK (STED)
B
i
ii

FigS4. BAX-BAK rings in human dermal fibroblasts.

(A) STED image of an apoptotic, fixed HDFa WT cell, immunolabeled for endogenous BAX (green, STED), BAK (magenta, STED) and TOM20 (blue, confocal). The cells were treated for 10h with 10 µM ABT-737, 10 µM Actinomycin D and 20 µM QVD-OPh.

(B) Enlarged insets from (A) showing mosaic BAX-BAK rings (arrow).

Scale bars: 5 µm (A), 1 µm (B).

FigS5

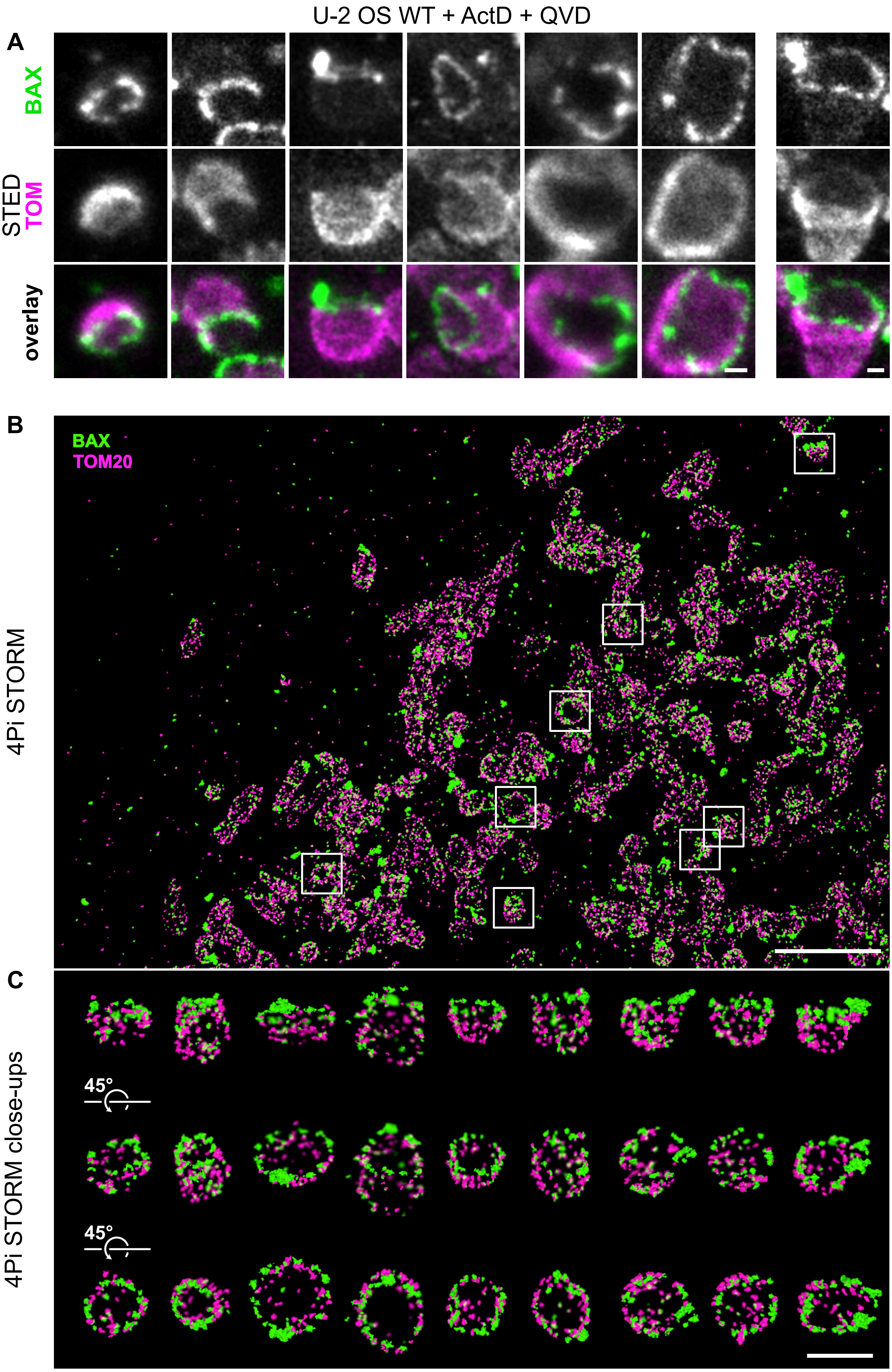
U-2 OS WT + ActD + QVD
A
BAX
STED TOM
overlay
B
BAX
TOM20
4Pi STORM
C
45°
45°
4Pi STORM close-ups

FigS5. BAX rings are devoid of the MOM protein TOM20.

(A) 2D STED images of apoptotic mitochondria from U-2 OS cells immunolabeled for BAX (green) and TOM20 (magenta) showing that the inside of the pore formed by BAX is largely devoid of TOM20.

(B) Representative 4Pi STORM image reconstruction of a 3D recording of U-2 OS cells immunolabeled for BAX (green) and TOM20 (magenta).

(C) Close-ups of single apoptotic mitochondria marked by rectangles in (B) displayed in 3 orientations. The inside of the pore formed by BAX (green) is largely devoid of TOM20 (magenta).

Scale bars: 200 nm (A-C), 5 µm (B).

FigS6

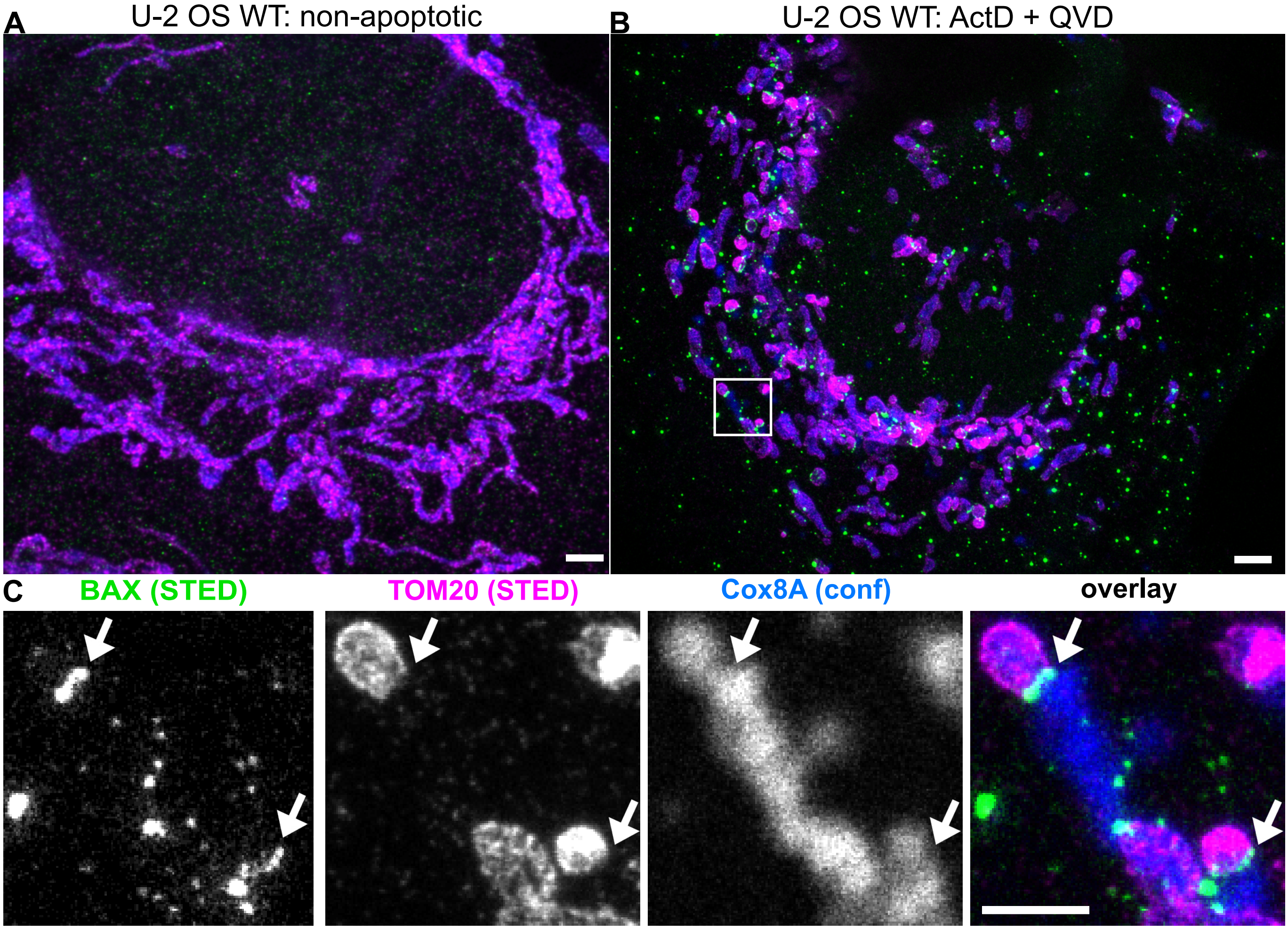
A
U-2 OS WT: non-apoptotic
B
U-2 OS WT: ActD + QVD
C
BAX (STED)
TOM20 (STED)
Cox8A (conf)
overlay

FigS6. MIM herniates through BAX rings.

(A) STED image of an untreated, fixed U-2 OS WT cell, stably overexpressing Cox8A-mNeonGreen (blue, confocal), immunolabeled for endogenous BAX (green, STED) and TOM20 (magenta, STED).

(B) STED image of an apoptotic, fixed U-2 OS WT cell, stably overexpressing Cox8A-mNeonGreen (blue, confocal), immunolabeled for endogenous BAX (green, STED) and TOM20 (magenta, STED). The cells were treated for 18h with 10 µM Actinomycin D and 20 µM QVD-OPh.

(C) Enlarged insets from (B). Arrows point at apoptotic rings, which release the mitochondrial inner membrane (Cox8A-mNeonGreen, blue).

Scale bars: 2 µm (A-B), 1 µm (C).

FigS7

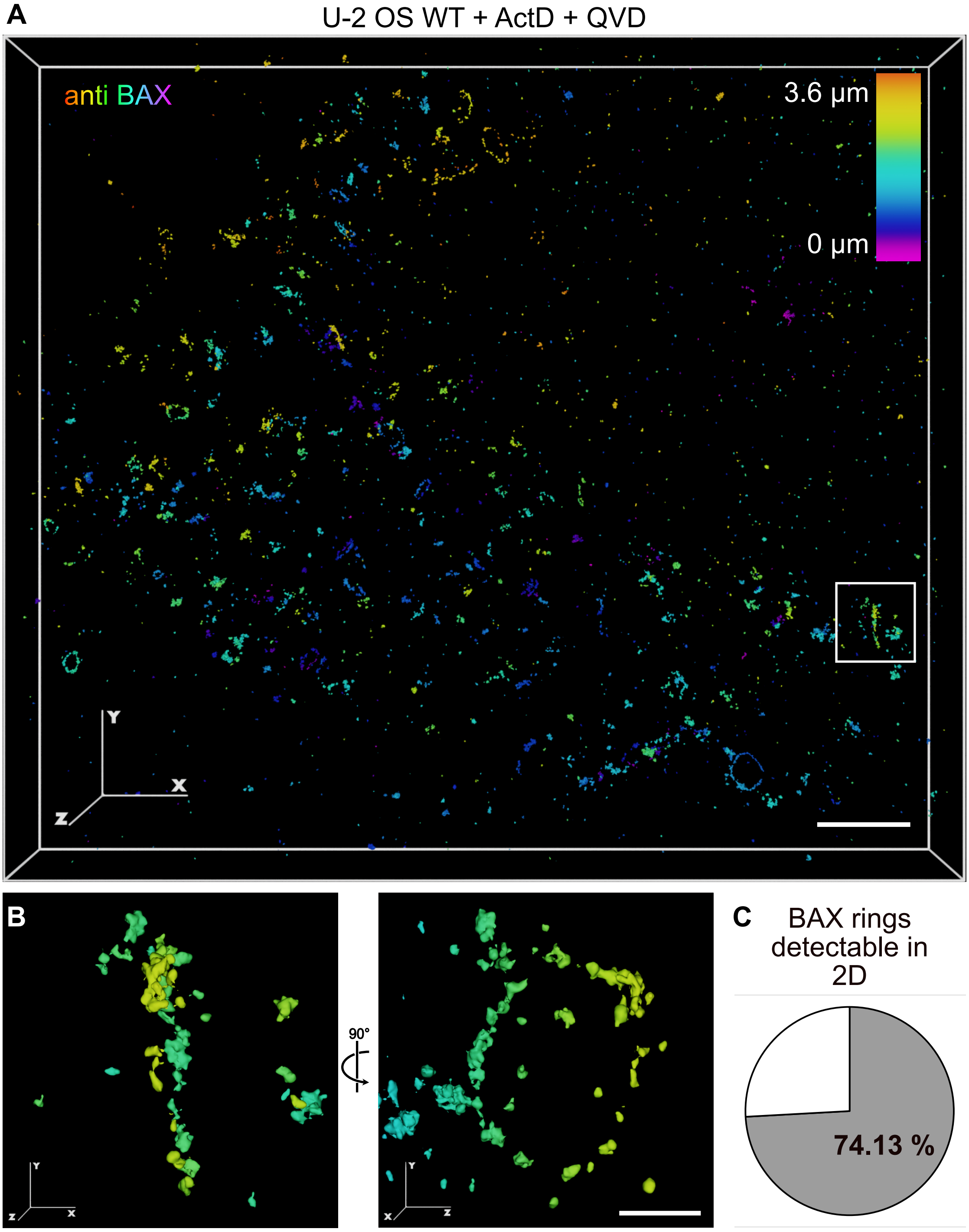
A
U-2 OS WT + ActD + QVD
anti BAX
3.6 µm
0 µm
Y
X
Z
B
90°
C
BAX rings detectable in 2D
74.13 %

FigS7. BAX rings are located in the cell in all spatial orientations.

(A) 4Pi-STORM image reconstruction of BAX in apoptotic cells. Data is displayed as perspective view along the z-axis with depth color coding. U-2 OS cells were treated with 10 µM ActD. 20 µM Q-VD-OPh was added to prevent the detachment of the cells from the coverslips. The apoptotic cells were fixed and labeled with the 2D2-BAX antibody, detected by a secondary Fab fragment coupled to Alexa Fluor 647, and prepared for 4Pi-STORM imaging. The image is a representative example of 3 replicates.

(B) Enlargement of the box in (A), as viewed from two angles. The left panel shows only a line of BAX, which is actually a BAX ring when turned by 90° (right panel). Note that in the front view of the ring (right panel) some BAX clusters appear to be inside the ring, but the side view (left panel) shows that these BAX clusters reside above and below the ring plane.

(C) Quantification of rings detected in 2D (gray area) vs 3D (full circle). Rings were counted in 5 datasets.

Scale bars: 2 µm (A), 250 nm (B).

FigS8

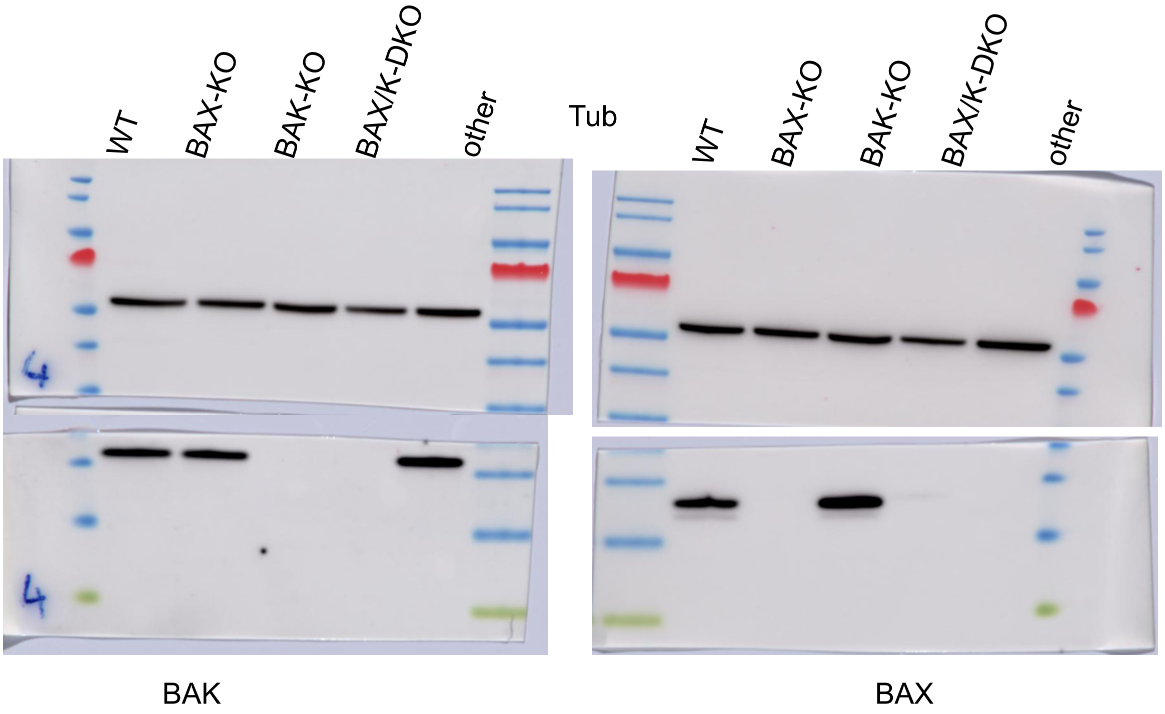
WT
BAX-KO
BAK-KO
BAX/K-DKO
other
Tub
WT
BAX-KO
BAK-KO
BAX/K-DKO
other
BAK
BAX

FigS8. Original Western Blot corresponding to Fig3A.

(Top, left and right) Tubulin as loading control.

(Bottom left) Anti-BAK.

(Bottom right) Anti-BAX.

Table 1: Immunofluorescence Antibodies

	Supplier	Cat#
Mouse anti human BAX Monoclonal Antibody (2D2)	ThermoFisher/invitrogen	# MA5-13994
Rabbit anti human BAX antibody, clone 1C7, ZooMAb® Rabbit Monoclonal	Merck/Sigma-Aldrich	# ZRB1103-4X25UL
Rabbit anti human BAK Recombinant Monoclonal Antibody (SU32-07)	ThermoFisher/invitrogen	# MA5-32111
Mouse anti human BAK antibody monoclonal [AT38E2]	abcam	# ab104124
Mouse anti human TOM20, purified, monoclonal, Clone 29	BD Transduction Laboratories	# 612278
Rabbit anti human TOMM20 antibody [EPR15581-39], recombinant, monoclonal	abcam	# ab186734
AffiniPure™ Sheep Anti-Mouse IgG (H+L) custom-labeled with Abberior STAR RED (Abberior # STRED-0002-1MG) or Alexa Fluor 594 (Thermo Fisher # A20004)	Jackson Immuno	# 515-005-062
AffiniPure™ Goat Anti-Rabbit IgG (H+L) custom-labeled with Abberior STAR RED (Abberior # STRED-0002-1MG) or Alexa Fluor 594 (Thermo Fisher # A20004)	Jackson Immuno	111-005-144
Mouse IgG anti-Chicken IgY (H+L)-unconj., MinX none	dianova	# SBA-8320-01
Rabbit Anti-Chicken IgY H&L	abcam	# ab97136
Rabbit anti human TOMM20 antibody [EPR15581-39], coupled to Alexa Fluor® 488	abcam	# ab205486
Alexa Fluor® 488 Mouse anti-Cytochrome *c* Clone 6H2.B4	BD	# 560263

Table 2: Western Blot Antibodies

Rabbit anti human monoclonal recombinant Anti-BAX antibody [EPR18284]	abcam	# ab182734
Rabbit anti human polyclonal Anti-BAK Antibody	Merck/Millipore	# 06-536
Goat Anti-Rabbit Peroxidase-AffiniPure IgG (H+L), Conjugation: Horseradish Peroxidase (HRP)	Dianova	# 111-035-144
Goat Anti-Mouse Peroxidase-AffiniPure IgG (H+L), Conjugation: Horseradish Peroxidase (HRP)	Dianova	# 115-035-062
Mouse anti-α-Tubulin antibody, monoclonal, clone B-5-1-2	Sigma	# T6074
